# Myocardial functional responses do not contribute to maximal exercise performance in the heat

**DOI:** 10.1186/s13728-015-0031-z

**Published:** 2015-07-24

**Authors:** Denise L Smith, Jacob P DeBlois, Margaret Wharton, Thomas Rowland

**Affiliations:** First Responder Health and Safety Laboratory, Department of Health and Exercise Sciences, Skidmore College, Saratoga Springs, NY 12866 USA; Saratoga Hospital, Saratoga Springs, NY 12866 USA

**Keywords:** Heat stress, Maximal exercise, Cardiac function

## Abstract

**Background:**

Both the extent and means by which maximal oxygen uptake ($\dot{V}{\text{O}}_{ 2\hbox{max} }$) is depressed by elevated ambient temperature are uncertain. Particularly, information is currently unavailable regarding the possible influence of alterations in myocardial function on $\dot{V}{\text{O}}_{ 2\hbox{max} }$ and performance during exercise in the heat. This study investigated the effects of environmental heat on $\dot{V}{\text{O}}_{ 2\hbox{max} }$, peak work capacity, and myocardial function during a standard, progressive cycle test to exhaustion. Twelve euhydrated men (aged 20.7 ± 1.7 years) performed a maximal cycle test in an environmental chamber in both heat stress [35°C, 30% relative humidity (RH)] and temperate (20°C, 30% RH) conditions with measurement of standard gas exchange variables, core temperature, and echocardiographic measures of cardiac function.

**Results:**

A small but statistically significant reduction of peak work capacity was observed in the heat stress versus temperate conditions (253 ± 30 and 259 ± 30 W, respectively, *p* = 0.02). Mean $\dot{V}{\text{O}}_{ 2\hbox{max} }$ was not statistically different in the two conditions (*p* = 0.16) but values were 3.4% lower in the heat, and 9 of 12 participants demonstrated lower values in the heat stress trial. No differences in responses of heart rate, cardiac output, stroke volume, core temperature, hydration status, or myocardial systolic or diastolic function were observed between the two conditions, but perceived body temperature was higher in the heat.

**Conclusions:**

The small, negative impact of heat on exercise performance and $\dot{V}{\text{O}}_{ 2\hbox{max} }$ could not be explained by disturbances in myocardial functional responses to exercise in young adult males.

## Background

Understanding the influence of ambient heat on human motor performance bears importance not only for athletes but health care providers in occupational and military medicine as well. Moreover, such information can be expected to provide insights into the basic physiological mechanisms which define exercise-induced fatigue.

Currently, research indicates that the effects of elevated environmental temperature on exercise capacity depend on the form of exercise being performed. During aerobic endurance exercise (i.e., events >30 min), elevations in ambient temperature clearly negatively influence performance in models of submaximal treadmill and cycle exercise time-to-fatigue [1, 2], as well as finish times in competitive race events [3].

This effect has been explained by a reduction in skeletal muscle perfusion caused by (a) a fall in plasma volume due to sweating-induced dehydration, and (b) a thermoregulatory “steal” of circulatory flow to the cutaneous circulation for convective heat dissipation from the skin [4, 5]. However, a review of recent studies indicates that reductions in endurance performance in the heat are observed in euhydrated participants without evidence of diminished muscle perfusion, supporting the alternative concept that elevations in core temperature trigger central nervous system responses which induce fatigue and limit exercise capacity [6].

On the other hand, individuals performing short-burst anaerobic exercise, such as cycle sprints of ≤30 s duration, typically demonstrate no negative effects of high ambient heat on performance [7–11]. Several studies have even indicated *improved* sprint performance in hot environmental conditions compared to thermoneutral [12–15].

This information suggests that the effects of elevated environmental temperature on motor performance relate to the *duration* of the high-intensity exercise being performed. Specifically, shorter exposure may preclude the time necessary for the development of dehydration and/or inhibitory rise in core temperature.

The extent that environmental heat dampens maximal oxygen uptake ($\dot{V}{\text{O}}_{ 2\hbox{max} }$) achieved during performance of a standard, progressive exercise test lasting 12–18 min is uncertain. Compared to thermoneutral conditions, reductions of 8 and 16% in $\dot{V}{\text{O}}_{ 2\hbox{max} }$ while exercising in the heat were reported by Sawka et al. and Nybo et al., respectively [16, 17]. González-Alonso and Calbet described an 8% reduction in $\dot{V}{\text{O}}_{ 2\hbox{max} }$ when participants were pre-heated with a hot-water jacket [4]. Both Arngrimsson et al. and Sakate demonstrated a direct relationship between environmental temperature and the magnitude of decrease in $\dot{V}{\text{O}}_{ 2\hbox{max} }$ [18, 19].

However, other studies have indicated no significant differences in $\dot{V}{\text{O}}_{ 2\hbox{max} }$ during progressive exercise protocols performed in hot and thermoneutral conditions [20, 21]. Schlader et al. recently reported similar levels of $\dot{V}{\text{O}}_{ 2\hbox{max} }$ when young, fit males cycled in 20 and 40°C ambient temperatures [22].

While a number of cardiovascular, thermoregulatory, and psychological variables might account for lower $\dot{V}{\text{O}}_{ 2\hbox{max} }$ in the heat, the explanation for such an effect remains uncertain. Several points of evidence suggest that alterations in myocardial function might play an etiologic role. Wilson et al. reported that in participants at rest, passive heat stress altered the Frank–Starling relationship and cardiac contractility, such that a minor fall in ventricular end diastolic (filling) pressure resulted in a large reduction in stroke volume [23]. Using tissue Doppler imaging, Fernhall et al. found a reduced lateral left ventricular relaxation rate in firefighters following a firefighting training exercise in hot conditions [24]. Stöhr et al. reported that cardiac function was affected by passive heat stress, but that mild exercise performed (knee extensions) with heat stress failed to trigger further increases in myocardial twist velocities, indicators of systolic and diastolic function [25].

However, reports of myocardial response to passive heat with participants at rest have consistently indicated *improvements* in markers of cardiac contractility [25–28]. The latter finding may be explained by concomitant decreases in preload and ventricular diastolic size in the heat, which influence measures of ventricular contractility [29].

No previous study has assessed myocardial systolic and diastolic functional changes during maximal exercise in the heat. The primary purpose of this study was to compare inotropic and lusitropic responses of young men to a progressive cycle exercise test designed to evoke $\dot{V}{\text{O}}_{ 2\hbox{max} }$ in temperate and heat stress conditions using standard Doppler echocardiographic techniques. Additionally, this study was designed to further examine (a) the effects of environmental heat on $\dot{V}{\text{O}}_{ 2\hbox{max} }$ and work capacity, and (b) the responses of other physiologic (core temperature, muscle blood flow, hydration status) and psychological (rating of exertion, thermal sensation) function which might contribute to any such effects.

## Methods

### Ethical approval

Written, informed consent was obtained from all participants prior to study procedures. All study procedures conformed to the standards set forth by the Declaration of Helsinki. The study was approved by the Institutional Review Board at Skidmore College.

Twelve normally active, college-aged men were recruited for this study. All participants reported exercising at least 3 days per week for 30 min per day during the previous 3 months. Exclusion criteria included known cardiovascular, metabolic and neuromuscular diseases or disorders, and history of smoking. Prior to testing, participants completed a medical history questionnaire and physical activity readiness questionnaire to screen for potential risks. Additionally, all participants underwent a medical evaluation by a health care professional and received medical clearance to participate. All participants were assumed to be non-acclimatized to heat, since the study was performed during the winter months in upstate New York.

This investigation employed a repeated-measures design. Participants performed a continuous graded exercise test to volitional exhaustion on a Monark Ergomedic 839 E cycle ergometer (Monark Exercise AB, Vansbro, Sweden) in an environmental chamber (Darwin Chambers Company, St. Louis, MO, USA) on two occasions under different environmental conditions: heat stress [35°C and 30% relative humidity (RH)] and temperate (20°C and 30% RH). Airflow velocity within the chamber was 15.2 ft min^−1^. Prior to testing, participants were familiarized with the protocol and testing devices as they cycled to at least 85% estimated maximal heart rate [220—age (year)] during heat stress. Conditions were presented in a balanced order. For each participant, trials were conducted at the same time of day and separated by at least 48 h. Participants were instructed to (a) refrain from strenuous exercise during the 24 h preceding testing, and to (b) consume 30 mL water kg^−1^ body mass, provided by the research team, the day prior to testing. Participants ingested a silicone-coated gastrointestinal core temperature capsule (HQ Inc., Palmetto, FL, USA) 6 h before the testing session for the measurement of core temperature. Prior to testing, urine specific gravity was assessed to confirm a euhydrated state (urine specific gravity ≤1.025). Nude body mass was obtained before and immediately following exercise testing using an electronic scale (599KL, Health-O-meter Professional, Alsip, IL, USA; accuracy ±0.1 kg). No fluids were consumed during the exercise trials.

A screening two-dimensional echocardiogram with Doppler velocity and color flow was performed prior to exercise with participants in the supine left lateral position to assure absence of structural or functional heart disease. All additional echocardiographic and Doppler studies pre- and post-exercise in this investigation were performed by a trained sonographer using an Aloka SSD model 5500 (Tokyo, Japan) with the participant seated on a cycle ergometer.

Prior to exercise, participants were seated on the cycle ergometer for 15 min in the environmental chamber to acclimate to the testing condition. Exercise was performed on an electronically braked cycle ergometer at a cadence of approximately 60 rpm. Initial power output was set at 60 W, and thereafter increased by 45 W every 3 min until exhaustion. Heart rate (Polar Electro Inc; Lake Success, NY, USA) and core temperature were recorded at each minute during exercise.

Rating of perceived exertion (RPE) [30] and thermal sensation (TS) [31] were obtained using standard visual scales during the last minute of each stage and at peak exercise. The RPE scale ranged from 0 to 10, with 10 corresponding to the highest level of exertion. The TS scale ranged from 0.0 to 8.0, with 0.0 designated as “unbearably cold” and 8.0 as “unbearably hot”.

Expired gases were collected throughout the protocol using a metabolic cart system (TrueOne 2400, Parvomedics, Sandy, UT, USA). The test was terminated when the participant indicated he could no longer continue or the tester determined that exercise cadence had dropped below the prescribed level.

Maximal oxygen uptake was defined as the peak rate of oxygen consumed (averaged over 15 s) during the final stage or last completed stage. Peak work capacity was calculated as watts achieved in the final completed stage plus watts pro-rated for any additional incomplete stages [time (s)/180 s × 45 W].

Heart rate was also measured electrocardiographically. Stroke volume was estimated as the product of the cross-sectional area of the aortic root (measured at rest) and the velocity–time integral (VTI) of blood flow, measured by continuous wave Doppler from the suprasternal notch using a 1.9 mHz Pedof transducer. The former was calculated from the diameter of the valve ring measured from the hinge points of the leaflets at maximal excursion in systole in the parasternal long axis view. Mean value of valve diameter was calculated from 3 to 4 consistent measurements. Beat-to-beat velocity–time curves were traced off-line to determine the mean VTI of the highest 3–8 consistent values.

Cardiac output (${{\dot{Q}}}$) was calculated as the product of heart rate (echocardiogram) and stroke volume. Cardiac output and stroke volume were indexed to body surface area as cardiac index and stroke index, respectively. Body surface area (BSA) was calculated based on the equation from DuBois and DuBois [32]. Arterial-venous oxygen difference, calculated as $\dot{V}{\text{O}}_{ 2} /\dot{Q}$, was assessed as a marker of skeletal muscle perfusion.

Mitral peak early diastolic inflow velocity (*E* wave) was recorded in the apical four-chamber view by pulse wave Doppler at the level of the tips of the open valve leaflets. Values were recorded as the mean of the highest 3–8 consistent velocity measures. Late diastolic velocity (*A* wave) was not considered, as this wave was observed to merge with the *E* wave beginning at low exercise intensities.

Pulse wave tissue Doppler imaging (TDI) was performed at the lateral aspect of the mitral valve annulus in the apical four-chamber view to record longitudinal left ventricular myocardial velocities in both systole (TDI-*S*) and diastole (TDI-*E*′). Transducer alignment was considered appropriate when the ventricular septum was vertical. Values were recorded and averaged off-line as the highest 3–8 consistently greatest velocities.

Echocardiographic images for this battery of variables were recorded at rest, beginning at 1 min and 30 s in each submaximal exercise stage, and during the final minute of exercise for determination of peak values. All recordings were obtained during spontaneous respirations.

Duration of ventricular ejection time was measured from the width of the aortic velocity–time curve. Peak aortic velocity was defined as the apex of the velocity–time curve.

Systolic myocardial function was assessed by peak aortic velocity, systolic ejection rate (stroke volume divided by systolic ejection time), and TDI-*S*. Diastolic function was examined by *E* peak velocity (reflecting upstream left atrial-to-downstream ventricular pressure gradient during early diastolic filling), TDI-*E*′ velocity (ventricular longitudinal myocardial relaxation properties), and *E*/*E*′ (left ventricular filling pressure). The measurement of *E*/*E*′ has been confirmed as a valid measure of left ventricular filling pressure in patients with heart disease, but its use in healthy participants is uncertain. Still, multiple studies in healthy participants have indicated that *E*/*E*′ consistently declines slightly in the course of progressive exercise [33–36].

These measures have all been well validated as reliable markers of left ventricular inotropic and lusitropic function [37–39]. Satisfactory levels of reliability for measurement of variables with this technique during maximal exercise testing have been reported previously, with coefficients of variation ranging from 2.8 to 8.1% at peak exercise [40, 41].

SPSS version 21 was used for statistical analyses (SPSS inc., Chicago, IL, USA). Results are expressed as mean ± standard deviation. Performance, physiological, and perceptual measures during the maximal tests were checked for normal distribution prior to statistical analysis. Anthropometric values and hemodynamic variables at rest and at maximal exercise were compared between the two conditions by a Student’s paired *t* tests. Mean difference scores between the two conditions were calculated as temperate − heat stress. The significance of changes in echocardiographic data during exercise was examined using a two-way ANOVA (condition × time) with repeated measures. Statistical significance was defined as *p* < 0.05.

## Results

**Figure 1:**
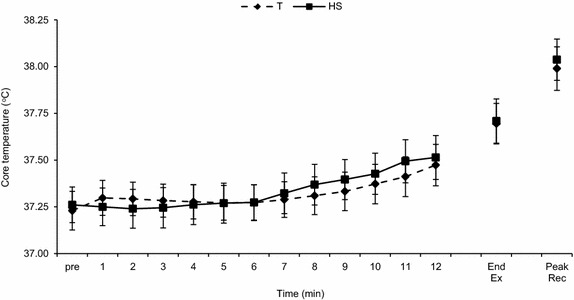
Core temperature during exercise by condition (*n* = 11). *Dashed line* temperate condition (T); *solid line* heat stress (HS) condition. Values are mean ± SEM. *End Ex* end exercise core temperature, *Peak Rec* peak recovery core temperature.

Screening echocardiograms revealed no evidence of cardiac disease in any of the participants. Anthropometric features of the participants and changes in nude body mass with exercise in the two conditions are presented in Table 1. Exercise produced no significant degree of dehydration in either the heat stress or temperate environment. During exercise, core temperature rose from 37.2 ± 0.3 to 37.7 ± 0.4°C in the temperate condition and from 37.3 ± 0.3 to 37.7 ± 0.4°C in the heat stress condition (no significant difference between conditions) (Figure 1).

**Table 1:** Descriptive characteristics of participants (*n* = 12)

Variable	Mean (SD)
Age (year)	20.7 (1.7)
Height (m)	1.78 (0.06)
Mass, baseline (kg)	82.8 (15.1)
Body mass index (kg m^−2^)	25.9 (3.7)
Body surface area (m^2^)	2.01 (0.20)
Body mass change, temperate (%)	−0.3 (0.3)
Body mass change, heat stress (%)	−0.5 (0.2)

Endurance time (*p* = 0.01) and peak work capacity (*p* = 0.02) were significantly greater in the temperate compared with the heat stress condition (Table 2), and these measures were higher in the temperate condition in 10 of the 12 participants (endurance time was 0.4 ± 0.4 min greater in temperate versus heat stress, while peak work capacity was 5 ± 7 W greater in temperate than heat stress). Mean $\dot{V}{\text{O}}_{ 2\hbox{max} }$ differences between the two conditions did not reach statistical significance (*p* = 0.16), but average values were 3.4% lower in heat stress (0.13 ± 0.30 L min^−1^ or 1.6 ± 3.6 mL kg^−1^ min^−1^), and 9 of 12 participants had lower values while exercising in the heat. No statistically significant differences were observed between temperate and heat stress environments for maximal heart rate or respiratory exchange ratio.

**Table 2:** Comparison of test time and maximal values of physiological data by condition

Variable	Temperate	Heat stress	Difference
Test time (min)	16.3 (2.0)	15.9 (2.0)^a^	0.4 (0.4)
Peak power (W)	259 (30)	253 (30)^a^	5 (7)
Maximal heart rate (bpm) (polar)	187 (8)	187 (6)	−1 (9)
End exercise core temperature (°C)	37.7 (0.4)	37.7 (0.4)	0.0 (0.4)
Rating of perceived exertion	9.3 (0.7)	9.5 (0.5)	−0.2 (0.7)
Thermal sensation	6.1 (0.8)	7.2 (0.7)^a^	−1.1 (0.8)
Oxygen uptake (L min^−1^)	4.07 (0.59)	3.93 (0.46)	0.13 (0.30)
Oxygen uptake (mL kg^−1^ min^−1^)	50.2 (8.7)	48.6 (8.5)	1.6 (3.6)
Respiratory exchange ratio	1.13 (0.05)	1.13 (0.06)	0.00 (0.07)
Arterial-venous oxygen difference (mL O_2_ L^−1^)	196 (40)	200 (43)	−4 (42)

Values are mean (SD).

*n* = 12 for all variables except for core temperature, where *n* = 11; difference calculated as temperate − heat stress.

^a^ *p* < 0.05 between conditions.

Ratings of perceived exertion rose similarly in both environmental conditions (Table 2). Thermal sensation was augmented in the heat stress condition compared to the temperate condition.

No significant differences in the peak responses of stroke volume, cardiac output, and arterial-venous oxygen difference were observed during exercise between the two conditions (Table 3). All measures of systolic and diastolic function were similar in the heat stress and temperate conditions at rest. The magnitudes of responses to exercise in each condition were comparable, and no significant differences were observed in values at maximal exercise. Markers of systolic and diastolic function rose by a factor of approximately two during exercise in both ambient temperatures.

**Table 3:** Myocardial function at rest and maximal exertion by condition

	Temperate (*n* = 12)			Heat stress (*n* = 12)		
	Rest	Maximal	Delta	Rest	Maximal	Delta
Stroke volume (mL beat^−1^)	81 (14)	116 (20)	35 (16)	72 (16)	111 (24)	39 (17)
Stroke index (mL beat^−1^ m^−2^)	40.3 (6.3)	57.5 (6.2)	17.2 (6.9)	35.5 (6.3)	54.9 (8.7)	19.4 (8.1)
Cardiac output (L min^−1^)	5.5 (1.1)	21.3 (3.5)	15.8 (3.0)	5.1 (1.3)	20.4 (4.1)	15.3 (3.3)
Cardiac index (L min^−1^ m^−2^)	2.7 (0.4)	10.6 (1.1)	7.8 (1.1)	2.5 (0.5)	10.1 (1.5)	7.6 (1.4)
Aortic velocity (cm s^−1^)	102 (10)	204 (23)	103 (21)	95 (10)	205 (29)	110 (23)
Ejection time (s)	0.247 (0.037)	0.173 (0.023)	−0.074 (0.025)	0.242 (0.034)	0.167 (0.015)	−0.075 (0.029)
Ejection rate (mL s^−1^)	326 (21)	673 (114)	347 (107)	296 (56)	661 (117)	365 (85)
*S* (cm s^−1^)	12 (2)	26 (3)	13 (3)	14 (2)	25 (3)	12 (4)
*E* (cm s^−1^)	69 (15)	167 (17)	97 (16)	71 (14)	159 (11)	88 (14)
*E*′ (cm s^−1^)	16 (3)	32 (5)	16 (7)	15 (3)	31 (4)	17 (5)
*E*/*E*′	4.4 (1.1)	5.3 (0.9)	0.9 (1.4)	5.0 (1.5)	5.2 (0.8)	0.2 (1.8)

Values are mean (SD).

Main effect of time (*p* < 0.001) for all variables except *E*/*E*′.

## Discussion

The central findings in this study were (a) among physically active young adult males, thermal stress from a high ambient temperature (35°C) effects minimal decrements in exercise performance, peak work capacity, and $\dot{V}{\text{O}}_{ 2\hbox{max} }$ on a standard, progressive cycle test to exhaustion compared to similar exercise performed in temperate environmental conditions, and (b) myocardial systolic and diastolic functional responses to maximal exercise are not influenced by a 15°C variation in ambient temperature and do not contribute to the differences in endurance exercise performance in this exercise model.

Endurance time was decreased by an average of 22 s (*p* = 0.01) when this group of 12 young men exercised in the heat compared to the temperate trial. Peak work capacity declined by 2.3% (*p* = 0.02), and $\dot{V}{\text{O}}_{ 2\hbox{max} }$ fell by 3.4% (*p* = 0.16). Peak work capacity was lower in the heat stress condition in 10 of 12 participants and $\dot{V}{\text{O}}_{ 2\hbox{max} }$ was lower in 9 of 12. Core temperature, as measured by a gastrointestinal pill, did not differ between trials in this short duration task. It is possible that esophageal temperature would have detected differences in body temperature as this method responds more quickly in response to exercise-induced heat stress [42].

Measures of both systolic and diastolic myocardial function in response to this acute bout of maximal exercise were similar in the two environmental temperatures. Similarly, no significant differences in maximal cardiac output and stroke volume were observed between the two conditions. Healthy individuals performing a progressive maximal exercise test normally demonstrate progressive increases in markers of both systolic and diastolic myocardial function with increasing work as a means of maintaining a stable stroke volume in the face of a shortening of ejection time (from increased heart rate). Typically, values such as left ventricular ejection rate and longitudinal tissue velocities rise in temperate conditions approximately twofold from rest to maximal exercise [43]. Our findings indicate that similar responses occur in both temperate and the moderate-heat stress conditions used in this study.

In the only other study that examined cardiac contractility changes during exercise in the heat, Stöhr et al. assessed systolic and diastolic function from rest to moderate (50% maximal) exercise using knee extensions (peak heart rate 134 ± 17 bpm) [25]. Echocardiographic measures of ventricular strain, strain rate, and twist dynamics recorded in a semi-recumbent position at rest revealed evidence of increasing systolic and diastolic function with three increasing levels of passive heat stress up to 38.5°C. However, these echocardiographic markers of myocardial function were unchanged by the exercise intervention. Furthermore, when exercise was added to the heat stress condition, there was no further change in these echocardiographic measures of myocardial function. Similarly, ventricular ejection fraction was greater with increasing levels of passive heat stress at rest, and values obtained during exercise and passive heat stress were not significantly different from heat stress alone.

The physiologic underpinnings for the small negative thermal effect on maximal exercise performance in this study were not evident by the parameters assessed. Core temperature rose similarly in both conditions by 0.4°C. No evidence was observed of diminished circulatory support to exercising muscle, as hydration status was maintained in both ambient temperatures, and there were no differences in maximal arterial-venous oxygen difference. No difference was observed in maximal respiratory exchange ratio in the two conditions, suggesting no impact of ambient heat on substrate utilization during the exercise bouts. It should be recognized that other potential contributors (such as direct measurement of muscle blood flow and regional blood flow) were not assessed in this investigation.

The only measure which differentiated exercise tests in the two conditions was the higher subjective report of thermal sensation while exercising in the hot environment. It might be reasonably suggested, then, that in the exercise model of this study, psychological factors might largely account for the minor decrements in exercise performance observed in the heat.

The nature of any potential psychological influence on physical performance in the heat was not elucidated in this study. In endurance events, reduced central drive as a protective mechanism against elevated core and brain temperatures has been considered as a limiting factor for performance [44], but core temperatures in the present study did not approach such levels, and changes in core temperature were similar in the two ambient conditions. Studies assessing the effects of ambient heat on mental function have provided conflicting results [45]. However, it is not unreasonable to suggest that, following common experience, the unpleasantness of exposure to high ambient temperatures might alter motivation to perform exercise.

The findings and conclusions in this study are congruent with the general concepts surrounding the negative influence of thermal stress on exercise performance. Increased ambient temperature reduces the thermal gradient between skin and surrounding air, impairing rate of convective heat loss and triggering a more rapid rise in core temperature as exercise proceeds [2, 46]. The latter, in turn, exaggerates sweat loss, which may accelerate the rise in core temperature as central blood volume falls, causing dehydration, which challenges both cutaneous blood flow for convective loss and perfusion of contracting muscle.

The *extent* of the thermoregulatory responses (sweating, convection) and the rate of rise in core temperature are thus key factors which determine the magnitude of negative impact on performance. By these mechanisms, the time spent in extended exercise in hot climatic conditions diminishes performance [44]. Shorter events, such as sprints, involve time durations insufficient to permit a rise in core temperature or dehydration, avoiding any negative impact on performance [8–10].

Findings in the present study, taken in context with other similar studies, suggest that thermal stress during a standard, progressive cycle test lasting 12–18 min may produce a negative impact on performance, representing an intermediate thermal challenge between endurance and short-burst exercise. Whether a small decrement in performance time (~2%) as seen in this study is significant for sporting events or occupational settings is unknown, as the ramped bicycle protocol used in this physiological study is not readily applicable to other settings. Furthermore, as the findings of Arngrimsson et al. suggest, the magnitude of the negative effect on performance may relate to the level of ambient temperature [18].

It should be recognized that this study involved a small number of participants, although it is reasonable to conclude that involvement of a greater number of participants to avoid type 2 statistical error would be unlikely to alter the conclusions of the study. In addition, the participant cohort was limited to healthy, nonobese, non-acclimatized, physically active, young males who were euhydrated prior to testing. The extent that the findings can be generalized to other groups and situations awaits further investigation. Furthermore, this study employed a singular bout of high-intensity exercise, whereas many sporting events and occupations require repeated bouts of high-intensity exercise in high ambient temperatures. Therefore, sports or occupations that require multiple bouts of exercise in the heat may exhibit different myocardial functional responses than those noted in the current study.

This study focused on markers of myocardial function, oxygen uptake, cardiac output, and core temperature during exercise. It should be recognized that these variables form only a portion of the myriad of circulatory factors that might be altered during exercise in the heat, including oxygen delivery in the face of maximized extraction, changes in muscle temperature flux, and regional alterations in cutaneous blood flow.

## Conclusions

High ambient temperature may result in depressed $\dot{V}{\text{O}}_{ 2\hbox{max} }$ in young, healthy men during a short cycling exercise bout (12–18 min). However, cardiac inotropic and lusitropic responses were not culpable for the reduction in oxygen uptake. In fact, all measures of physiological responses remained similar between the same exercise bout in high (35°C, 30% RH) and temperate (20°C, 30% RH) ambient conditions. Instead, psychological factors may play a role in exercise performance. These conclusions are tempered by the fact that physiological parameters measured in this study did not include all those which might potentially account for differences in exercise performance.

## Availability of supporting data

The data set supporting the results of this article is included within the article.

## Abbreviations

$\dot{V}{\text{O}}_{ 2\hbox{max} }$: maximal oxygen consumption
RH: relative humidity
RPE: rating of perceived exertion
TS: thermal sensation
VTI: velocity–time integral
$${{\dot{Q}}}$$: cardiac output
BSA: body surface area
TDI: tissue Doppler imaging
